# Overcoming bias in the comparison of human language and animal communication

**DOI:** 10.1073/pnas.2218799120

**Published:** 2023-11-13

**Authors:** Erica A. Cartmill

**Affiliations:** ^a^Department of Anthropology, University of California, Los Angeles, CA 90095; ^b^Department of Psychology, University of California, Los Angeles, CA 90095

**Keywords:** ostension, inference, meaning, gesture, human uniqueness

## Abstract

Human language is a powerful communicative and cognitive tool. Scholars have long sought to characterize its uniqueness, but each time a property is proposed to set human language apart (e.g., reference, syntax), some (attenuated) version of that property is found in animals. Recently, the uniqueness argument has shifted from linguistic rules to cognitive capacities underlying them. Scholars argue that human language is unique because it relies on ostension and inference, while animal communication depends on simple associations and largely hardwired signals. Such characterizations are often borne out in published data, but these empirical findings are driven by radical differences in the ways animal and human communication are studied. The field of animal communication has been dramatically shaped by the “code model,” which imagines communication as involving information packets that are encoded, transmitted, decoded, and interpreted. This framework standardized methods for studying meaning in animal signals, but it does not allow for the nuance, ambiguity, or contextual variation seen in humans. The code model is insidious. It is rarely referenced directly, but it significantly shapes how we study animals. To compare animal communication and human language, we must acknowledge biases resulting from the different theoretical models used. By incorporating new approaches that break away from searching for codes, we may find that animal communication and human language are characterized by differences of degree rather than kind.

Human communication draws on highly developed ostensive and inferential capacities (1–3). While much of language is codified and structured, humans can use virtually any behavior to communicate (e.g., a head tilt, a look, moving an object). The ostensive capacity to turn anything into a signal is seen as a powerful driver of human communication. The hypothesized mechanisms underlying this capacity have become one of the last bulwarks of human language’s uniqueness.

There is a long history of searching for the property that makes human language unique. Many properties once considered unique to humans (e.g., referential communication, syntax, cultural transmission, and intentionality) have subsequently been found in animal communication systems, at least in simple forms. I argue that recent claims that the cognitive foundations of human language are unique are not supported by the evidence cited, because the theories and methods used to study human language are not comparable to those used to study animal communication. These differences bias the conclusions we draw about the nature of the two systems.

## Searching for Features Unique to Human Language

Human language was once thought to be a more formal, structured system, in contrast to the emotional, contextually bound communicative acts of animals. Even Darwin’s famous work comparing the expression of emotions in humans and other animals set “articulate language” to one side, considering it to be an altogether different type of system (4). These perceptions were influenced by a long tradition in Western philosophy privileging humanity’s capacity for rational thought, as well as by models of language that focused on our ability to convey meaning in predictable, decontextualized ways (5). These models were often developed by people studying written language and accordingly focused on the structural features and decontextualized, disembodied aspects of language.

For most of the 20th century, scholars argued that structural features of human language distinguished it from animal communication. The search for the structural “key” to language turned into an arms race between linguists and animal communication researchers. Every time a linguistic feature was proposed as the Rubicon separating human language and animal communication, animal researchers would look for evidence of that ability in their study species. Eventually, it would be found in one or more species (though in simpler or attenuated forms). In order to demonstrate the ability in nonhuman animals, researchers would often broaden its definition to make sense for nonlinguistic communication. This sometimes changed the definition considerably. Linguists would object. Animal researchers would defend their generalizations. This cycle was repeated many times.

Four of the primary candidates for features differentiating human language and animal communication—cultural transmission, reference, syntax, recursion—are reviewed below. Many other aspects of communication have been compared between animal and human systems (e.g., turn-taking, multimodality, intentionality), but these four illustrate how diverse aspects of language—learning, meaning, and structure—have been identified in animal systems. Note that finding a feature of human language in an animal system does not imply that language is not unique. Each feature has been found in one or more animal species, but never to the degree seen in human language. Language supercharges these features, and it is likely unique in its constellation thereof.

Cultural transmission was long assumed to be a defining feature of human language since language is acquired through convention and culture rather than biological inheritance. We now know that many other species, particularly birds, also acquire their vocalizations through learning from adult models. Some species even have a “critical period” in infancy, during which they learn from song models and rehearse their songs, and after which they cannot learn to produce these songs correctly (6). Evidence of vocal learning coupled with evidence of dialect differences across populations led scholars to conclude that there was cultural transmission of vocalizations in birds, whales, bats, and elephants (7–12)^*^.

Reference (using a signal to denote something in the external environment) was once argued to be unique to human language. Then, researchers demonstrated that the alarm calls of many types of animals differ systematically in response to different types of predators and elicit different escape behaviors in listeners (13, 14). This predictable association with an external event seemed to meet the broad definition of reference; however, animal researchers began to use the term “functional reference,” since no one knew whether the animals intended to refer to the predators when producing alarm calls. Once animals appeared to demonstrate a capacity for (at least functional) reference, displaced reference became the new bar. Humans not only refer to things in their immediate environment but also refer to things displaced in space or time (5, 15). In contrast, the animal alarm calls that demonstrated functional reference were always produced in the presence of a predator (the referent). Arguments for the uniqueness of displaced reference dovetailed with arguments that only humans were capable of mental time travel: the cognitive ability to remember the past or imagine the future from a first-person perspective (16). Experimental paradigms have since shown that great apes can point to things in their immediate environment to reference objects displaced in space and time (17). Other studies suggest that apes and other species are capable of mental time travel; they plan for future opportunities to retrieve food by selectively caching different types of food or storing tools where needed to retrieve future rewards (18–20).

Many scholars have argued that syntax is the defining feature of human language (21–24). Syntactic rules allow humans to communicate about an infinite number of things with a finite number of symbols. Having a set of rules that can be applied in predictable but open-ended ways to a set of symbols (e.g., words) enables humans to create sentences that they have never heard before. No other species has been found to use syntax as humans do, but evidence of syntax-like structural rules has been found in several animal communication systems. The songs produced by many bird species have “rules” that predict which sounds can be combined into motifs and which motifs can follow one another (25, 26). The same is true for whales (27) and the call sequences of some primates (28). When perception of song sequences is tested by playing back recordings to birds, most species recognize when components are presented in orders that break the rules (29–31); there is little evidence, however, that rules about component order encode differences in meaning. Some argue that the “syntactic” patterns in bird and whale song are better described as phonological rules (or phonological syntax rather than lexical syntax), since they apply to sound combinations but not, as far as we can tell, to changes in meaning (25). Note that I use meaning throughout this paper to encompass both the rich, intended meanings of human language (and arguably ape gestures), and the information receivers extract from animal signals.

Animals can combine signals to amplify or modify one of the component parts (32–37)—for example, by increasing intensity. However, there is little evidence that signal combinations produce meanings that differ from their parts. This type of combination (A + B = C) would suggest a syntax more reliant on the rule than on the identity of the components, one where the combination takes on an entirely new meaning. Such rules have been hinted at in the call sequences of a few monkey (38–40) and bird species (41, 42), but not all scholars are convinced these systems require syntax to explain the observed patterns (43). Of course, animals might combine calls for reasons that have nothing to do with changing the meaning of the component parts. Song complexity has been linked to reproductive success across different taxa, suggesting that the ability to combine different sounds is itself attractive (44, 45).

One particular type of syntactic rule—recursion—has been proposed as the feature that distinguishes both human language and human minds (46). Recursion is the ability to apply a syntactic rule to the output of that same syntactic rule, ad infinitum. This enables humans to create infinitely long utterances with multiple embeddings. For example, the utterance “I read the sentence that Cartmill wrote that illustrated the property that Chomsky described that Fitch argued made human language unique” exhibits several instances of recursion. This type of rule is complicated to employ and difficult to test, but one study found evidence that European starlings are capable of distinguishing patterns that rely on recursion (47). Others have argued that these results may have been based on numerical cognition rather than understanding of recursion (48), and so the search continues.

In recent years, the focus on structure as the defining aspect of language has relaxed. Indeed, the broad characterization of human language as structured and of animal communication as lacking structure has largely reversed. Human language is now often characterized by its flexibility, openness, and ability to deviate from its “code,” while animal systems are depicted as inflexible and reflexive, restricted to operating within set rules (3, 49, 50). This shift from structural features to cognitive building blocks aligns with a broader shift towards studying the minds rather than just the behavior of animals (51). However, shifting from the features of communicative acts to the often invisible motivational states and cognitive abilities behind them makes it more difficult to draw comparisons across species. As we shall see, the shift toward meaning and cognition also gives theoretical models of communication—and their methodological implications—an outsized role in shaping the empirical evidence about animal abilities.

### Human Language Relies on Understanding Others’ Minds.

Human language is an exceptionally rich communicative system, even when it appears in the impoverished form studied by most linguists (written down and stripped of its nonverbal components and interactional information). Sociolinguists and linguistic anthropologists argue that human language is characterized not by the formal features seen in disembodied and decontextualized examples but by the rich social cognition and pragmatic inferences that underlie face-to-face communication. Pragmatics is the branch of linguistics that focuses on how language is used in everyday life and how meaning is conveyed in situated, interactional contexts rather than through only words and syntax. The heart of pragmatics is the idea that language meaning is inflected by its physical, social, and conversational environments.

The flexibility of human language means that each language user has the potential to modify, embellish, or expand the system, either in the long term (by inventing new words or structures) or in the short term (by using things outside the linguistic system in communicative ways). Long-term modifications allow languages to change over time. Short-term modifications allow speakers to adapt particular communicative acts to their goals, interlocutors, and environments. This might involve weaving together conventional symbols and structures with nonverbal elements like gestures, facial expressions, intonations, or prosodic emphasis. It may simply involve rich inferences about the goals or knowledge of one’s interlocutor. Take, for example, the following exchange between two friends at a party:

1. Susan: Is George here?
2. Lila: I saw an orange truck when I came.

To understand this exchange, you do not need to know that George drives an orange truck; you can extract the meaning from the response. This is done through inference, rather than a literal reading of the sentence. Many human conversations are intelligible only because (A) the speaker makes clear that their actions are communicative and (B) the listener makes a series of inferences about the relevance of the speaker’s actions to the ongoing conversation. The shared goals and coordinated actions of speaker and listener are encapsulated in the cooperative principle of philosopher Paul Grice (52). It is debated whether animal communication in general—and great ape gestures in particular—ever display Gricean principles of communication (53–57).

The cognitive abilities underlying our capacity to communicate in novel, nonliteral, and flexible ways are ostension and inference. Through ostension, a person draws attention to their actions and conveys that they are communicative (e.g., sliding their glass towards someone who is pouring wine). Through inference, others can perceive the communicative intent and respond accordingly (e.g., by filling the glass). The ostensive-inferential model of human language proposes that signalers are concerned with making their communicative intentions known (and may use a variety of behaviors to provide evidence of intention), and listeners are concerned with gathering this evidence and making inferences about the intended meaning (1, 3, 49, 58, 59). In line 2 of the example above, Lila answers Susan’s question by referring to an unusual type of car (not to George). Susan makes sense of this reply by assuming that Lila’s utterance was relevant to her question and infers that George is present. Such indirect reference seems complicated, but it is common in everyday language and demonstrates how easily humans employ ostensive-inferential abilities. Importantly, it relies heavily on understanding others’ minds and intentions: so-called theory of mind (2). There is evidence from cognitive studies that animals possess at least some aspects of theory of mind (e.g., understanding gaze), but the ways in which we typically study animal communication do not leave much room for questions about ostension and inference.

### Socio-Cognitive Abilities in Animals.

Social animals have evolved sophisticated cognitive abilities that help them navigate their social worlds. These abilities include learning from others, tracking relationships across time, predicting others’ actions, making inferences about others’ goals, and responding to others’ behavior based on inferences about what they can or cannot see refs. (60–62).

Nonhuman primates are renowned for complex social cognition, which they use to navigate social hierarchies, form friendships, recruit allies, and avoid rivals (63–65). They understand not only their own relationships, but the nature of relationships between others (66). There is evidence that at least great apes understand what others can and can’t see ref. (60), as well as what others know and don’t know (67, 68), suggesting that they possess some type of theory of mind. Adult humans can understand long recursive chains of theory of mind (e.g., I know that Matt knows that Jacob believes that Gal hopes that I want to see the movie tomorrow), but the building blocks of understanding other minds develop slowly over the first few years of life (69, 70). Human toddlers and great apes both pass simple theory-of-mind tests; for example, by visually predicting where others will search for an item that the subject knows has been moved (65, 67, 71, 72). Note, however, that young children (but not apes) have at least some knowledge of language, which may give them an advantage in propositional thinking about others’ beliefs (73).

Some argue that socio-cognitive abilities like theory of mind provide the foundation underlying human language but only enrich (rather than underlie) animal communication (1, 3, 49, 50). This might seem like a semantic quibble, but the difference is significant. The claim is that human language is built on a fundamentally different type of cognition (one that requires ostension and inference), while all animal systems are exchanges of codes that do not require inferences about others (but may sometimes employ them). This model of language origins argues that language is not an animal-like system enriched with properties like syntax and complex inferences about others’ intentions. Instead, it claims that language is an entirely new type of system reliant on inferences about others’ minds at its most basic level. This claim is intriguing but it proposes that human language represents a discontinuity in the evolution of animal communication systems: Language involves sophisticated social inferences about signaler intentions and recipient knowledge purportedly lacking in animal systems. This, in turn, requires considerable evidence that rich social inferences about others’ minds are missing from nonhuman communicative acts. The difficulty is that data on human and animal communication have been heavily biased by differences in the way humans and animals are studied.

### Animal Communication As Codes.

**Fig. 1.**
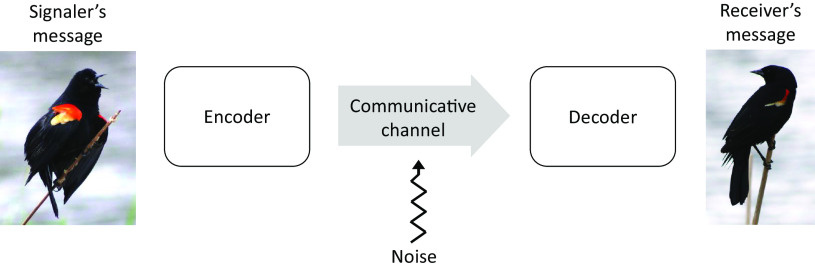
A schematic illustrating the code model of communication.

Animal communication research has been strongly shaped by the “code model” of communication, though it is rarely referenced directly (Fig. 1). In this model, a message is encoded by the signaler, transmitted across a communicative channel (where it can distorted by “noise”), and then decoded by the receiver. The goal is for the decoded and encoded messages to be the same, with little information lost during transmission. This model grew out of Shannon’s information theory (74) and the conduit model of information transfer (75). The code model reflects its origins—the transmission of electronic signals in broadcasting and engineering—through the metaphors of encoding, decoding, transmission, noise, and signal interference.

The code model provides a useful recipe for breaking communication into phases, each a potential source of miscommunication. For example, a signaler could choose the wrong signal, the signal could be difficult to perceive due to interference (noise), or the receiver could misinterpret the signal and respond inappropriately. In describing potential sources of miscommunication, I have avoided code model terms (encoding, decoding, error, transmission) because they equate communication with robotic transfer. Herein lies the main problem with the code model: It dramatically restricts the ways we conceptualize (and study) animal communication.

The code model has had a pervasive effect on the design and interpretation of research on animal communication. Studies are predominantly built around searching for replicable contingencies between a signal produced by one individual and a subsequent change of behavior in another—in other words, codes (76). This search for code-like systems is mostly unavoidable; it is the primary way researchers demonstrate that animal signals have meaning (i.e., that they are communicative). If a signal does not reliably relate to a feature of the environment and/or evoke a particular response, then it’s hard to argue that it means anything—at least if your starting assumption is that animal signals are meaningless until proven otherwise.

This approach differs profoundly from how meaning is studied in language. Many human words, sentences, and gestures do not evoke an immediate reaction in others. Consider the paper you are currently reading. Unless reading this paper led to a predictable response in readers (e.g., angrily throwing it across the room or immediately designing a new study), standard methods in animal communication would conclude that the paper had no meaning. Methods for determining meaning in animal signals are not good at detecting communicative acts with delayed reactions, acts that impact internal states rather than external behavior, or acts that are embedded within ongoing interactions and are difficult to isolate.

### Methodological Bias in the Search for Meaning.

To establish that animal signals have meaning, researchers focus on isolated signals that elicit predictable, immediate behavioral reactions. This makes sense; humans are not fluent in the systems they are studying, and animals cannot provide translations. However, this focus on predictable contingencies between signals and responses tightly constrains how researchers study meaning in animal systems and which behaviors they study in the first place.

The gold standard for determining that an animal signal has meaning is the “playback” design. There are many forms of meaning-making that draw on social, contextual, or environmental features outside of the signal, but the playback method looks only at the meaning transmitted to the receiver by the information encoded in the signal itself (directly borrowing metaphors from the code model). In a playback design, a signal (almost always auditory) is recorded and later played back to one or more animals to observe their reactions. It is critical to play the recorded signal back outside of its original context so that the environmental conditions that elicited the signal are not present; any responses will then be due to the information in the signal.

Playback designs are effective at measuring particular types of signals (alarms), in particular modalities (auditory), with particular responses (immediate behaviors like fleeing). With careful experimental manipulation, the playback method allows researchers to test their attributions of signal meaning. This is a major advantage. Humans bias our ascription of meaning to animal signals by the ways we parse and label our worlds. Alarm calls are typically thought to reference the type of predator that elicits them (e.g., leopard, eagle), but animals might perceive and categorize the world differently. For example, domestic chickens were thought to have separate alarm calls for foxes and eagles. When chickens heard an eagle alarm call, they hunched down. When they heard a fox alarm call, they flew up to perches. However, when chickens were shown video of foxes on screens overhead, they gave “eagle” calls. And when they saw videos of eagles on the ground, they gave “fox” calls (77). This simple manipulation revealed that chickens do not have alarm calls referencing the type of animal [something humans, including infants, would consider a “natural kind” (78, 79)]; instead, they provide information about the location of the impending attack.

Studies of dynamic visual signals (like gestures) also look for contingencies between signals and responses, though it is usually not possible to play back visual signals. In animal gesture studies, researchers typically take all cases of a particular gesture form, filter for those with markers of intentional use, categorize and count receivers’ responses, and assign meaning to gesture types based on apparently satisfactory outcomes—situations where the gesturer ceases to signal rather than persisting (80–82). After finding that signalers do not continue gesturing following Response X to Gesture A—but do persist following other responses to Gesture A—researchers conclude that Gesture A likely means “do X”. Such analyses of meaning focus on the signaler’s goal (what response from the receiver satisfies the signaler). This differs from playback studies, where analyses of meaning focus on how receivers perceive, interpret, and respond to the signal. This perspective shift is important because the goals of communication differ significantly between signalers and receivers (83). Analyses of gesture meaning and auditory playback designs are both successful at studying signals used in isolation with clear, immediate, behavioral responses, but this represents only a subset of all potentially communicative behaviors. What happens to other behaviors?

Animal signals not reliably associated with particular responses are often dismissed as “ambiguous” and excluded from analyses of meaning (or structure). In this way, studies present a highly curated subset of all potentially communicative behavior. This curation allows researchers to focus their efforts on frequent actions with immediate, predictable responses. Focusing on a subset of behavior has the added benefit of increasing the sample size for analyzed signals and standardizing methods across studies.

The use of playback to study alarm calls has become the paradigm for rigorously demonstrating that animal signals have meaning. Indeed, most studies of meaning in animal communication use variations of the playback design and draw directly on metaphors from the code model, like signaler and receiver. However, this limits the kinds of questions researchers can ask and the conclusions they can draw.

### The Code Model Constrains Even Studies of Flexible Animal Systems.

Not all kinds of communication can be studied via playback, but methodological practices stemming from the code model skew conclusions about these systems as well. To illustrate this, I draw on examples from ape gesture. I do this for two reasons: First, because I have worked in this field for almost 20 y, so any criticisms I make necessarily include my own work; second, because ape gesture is arguably the least likely animal system to fall prey to the constraints of the code model. From the start, ape gesture has been recognized as both intentional and flexible—properties that make it more similar to human communication and significantly less code-like than other animal systems (84–89). This focus on intentionality and flexibility helped to distinguish ape gestures from vocalizations, which were argued to be largely inflexible and contextually bound (90, 91). Ironically, it was only once ape gestures had been shown to be used flexibly that researchers began to systematically investigate gesture meaning by looking for tighter relationships between gestures and responses (i.e., codes). By highlighting the ways that the code model permeates study designs of even such extremely flexible and intentional animal systems as ape gesture, I hope to show how pervasive the search for codes is in studies of animal communication.

To study gesture, researchers start with video of ape groups. They cut these videos down to focus on nonfunctional movements produced in social situations, excluding movements produced when apes are alone and instrumental movements that achieve a physical goal (like picking up an infant). The remaining movements are categorized by their physical forms and contextual uses. They are often named by their similarity to human gestures (e.g., beckon, shoo, wave). Researchers may exclude gestures that do not meet criteria for intentionality, like waiting for a response or elaborating when there is no response (92). It is also common to exclude gestures that occur during play or multiparty interactions, since these are not likely to occur the same way multiple times. Most studies also exclude “rare” gestures observed only infrequently. Sample size is particularly important for analyses of meaning or function, where many examples of the same gesture are needed. In studies with smaller sample sizes, rarely observed gestures may be a sizable portion of the data. For example, Cartmill & Byrne (80) described 64 gestures in orangutans but included only 40 in analyses of meaning. Genty et al. (93) reported 102 gestures in gorillas but analyzed function for only the 10 most common. Finally, analyses of gesture typically focus on gestures that either occur alone or at the start of sequences, i.e., those that initiate interactions. This increases the likelihood that a gesture can be shown to elicit a particular response, critical for demonstrating that gestures have (code-like) meanings.

These curation and analysis practices have led to important discoveries about ape gesture. Nevertheless, it is important to recognize what these practices leave out and to recognize the ways in which they too have been shaped by assumptions of the code model. Table 1 presents examples of findings and methods from a range of ape gesture studies. This table is by no means exhaustive; it is meant to demonstrate how pervasive the search for codes is, even for a type of communication characterized by flexibility and intentionality. Some current studies of ape gesture attempt to move beyond searching for code-like contingencies by focusing on the pragmatics of interactions or socio-ecological contexts of communication (94–99); I discuss these below. Despite recent work to widen our lens, the study of ape gesture continues to be blinkered by the code model, in both methods and research questions.

**Table 1.** Comparison of methods and conclusions from published studies of ape gesture meaning (emphasis mine)

Study	Species	Conclusions	Methods
Hobaiter & Byrne (85)	Chimpanzee (*Pan troglodytes*)	“…found [gestures] to be used intentionally to achieve 15 purposes...”	“We examined whether different gestures were associated with a specific pattern of outcomes..” “… we therefore excluded data from play bouts to avoid masking the “real-world” meaning of gestures.”
Roberts et al. (104)	Chimpanzee (*Pan troglodytes*)	“...gestures were also strongly associated with specific responses and outcomes…”	“…consistent association between a given gesture type and particular behavioural change may be used to infer the meaning of different gestures” “…this necessarily excluded both actions that were not clearly directed…towards a specific recipient that could visually perceive the signaller's behaviour…and actions that could feasibly be readily explained in noncommunicative terms”
Cartmill & Byrne (84)	Orangutan (*Pongo pygmaeus/abelii*)	“…more than half of the orangutan gestures we were able to analyse had predictable intentional meanings”	“The aim is to identify gestures that are used predictably to elicit specific reactions...” “Only gestures that occurred singly or as the first gesture in a sequence were analysed for meaning. While this simplified the analysis by restricting it to a single signal and reaction in each case, it necessarily excluded some gestures from analysis.”
Graham et al. (105)	Bonobo (*Pan paniscus*)	“Bonobos intentionally deploy gestures to achieve at least 14 different intended outcomes”	“We are able to deduce the meaning of great ape gestures by looking at the ‘Apparently Satisfactory Outcome’ (ASO)…” “…Fifteen gesture types were suitable for analysis, having been used by at least 3 individuals at least 3 times to achieve an ASO.”

These studies represent a range of authors and study species and all employ methods frequently used in the field. Each study found that gestures were used to communicate specific meanings. Each also explained in the methods that the researchers were explicitly looking for code-like contingencies between signal and response and curated their data in particular ways that made these codes easier to find.

### Meaning beyond Codes.

Studies of animal communication (like those in Table 1) typically find that animals communicate using simple codes that elicit particular responses. However, this is unsurprising: These studies are looking for codes, often discarding communications that do not fit the code model.

Human language, in contrast, is already assumed to be both communicative and meaningful. This assumption frees up the ways it is studied. Flexibility in the way words and gestures are used is assumed to reveal properties of thought or interaction, rather than evidence that the observed behaviors are not communicative. A word doesn’t need to show a predictable response in order to be considered meaningful.

**Fig. 2.**
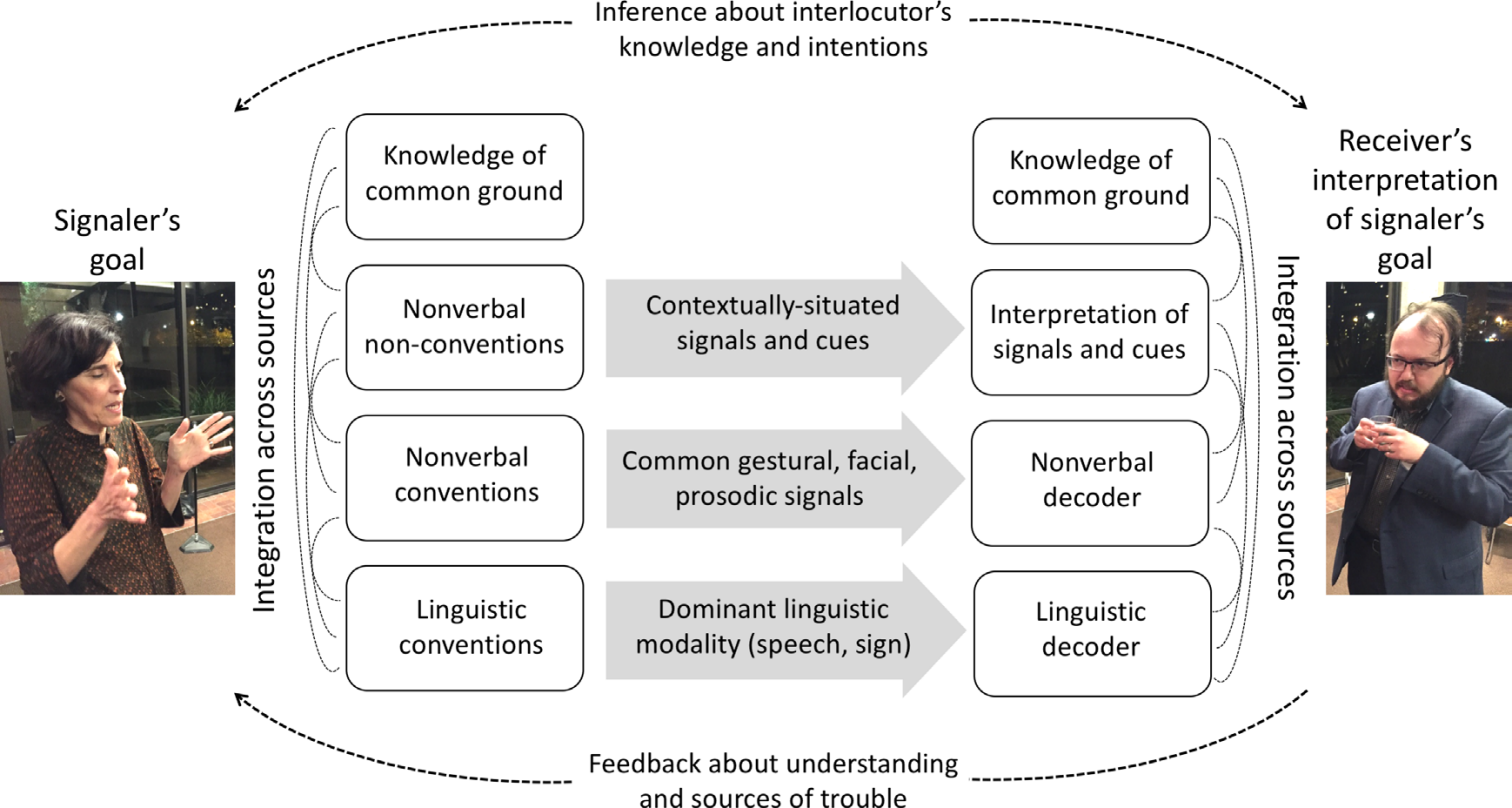
Schematic illustration of some of the sources of meaning humans integrate when communicating. Sources with code-like features (e.g., words) form only a small part of the semiotic landscape in natural conversation.

Humans communicate by integrating and interweaving culturally transmitted codes (spoken or signed language) with nonverbal conventions (e.g., gestures, prosody), nonverbal nonconventions (e.g., interacting with the physical environment), and assumptions about the knowledge and goals of their interlocutor. This integration occurs for both signaler and receiver, with information exchanged in both directions. Receivers provide feedback when they encounter difficulties in understanding, and signalers revise their communicative acts in real time based on this feedback (100, 101). Studies of human gesturing demonstrate that gesture is tightly integrated with speech, both semantically and temporally (102–104). When one modality is disrupted (as in the case of stuttering), the other is as well (105, 106). Receivers also integrate information across gesture and speech when interpreting language (107, 108). Evidence from studies of gesture production and gesture perception supports a model of human language as a single, integrated, multimodal system—rather than two systems produced at the same time (109–112). We have only begun to understand the full range of behavior and information humans use when communicating face-to-face. Fig. 2 gives an overview of some of the many possible sources of meaning humans draw on.

Fig. 2 is not a comprehensive model of human communication (113); it merely highlights the many ways humans convey meaning to one another. Note, however, how complex this simplified model of human communication is when compared to the code model used to study animal communication. Humans might ostensively signal that they are communicating by drawing on a wide range of sources: linguistic conventions, nonverbal conventions, or nonverbal nonconventions. For example, imagine that the people in Fig. 2 are at an event when the music switches to an obscure song. The signaler might smile and hold one finger up to draw attention to the song (nonverbal conventions), say “I remember digging through records at an estate sale and finding this waaay in the back,” and mime flipping through record albums (a nonverbal nonconvention). The receiver integrates information across the speaker’s words, actions, tone, expression—and the song itself—with inferences about the speaker’s intentions to interpret the communicative act.

It is unlikely that such a complex personal narrative would be found in a nonhuman animal. However, it seems possible (and even likely) that at least some animals use some of these ostensive and inferential abilities at least some of the time. This is especially true for great apes, who flexibly modify their gestures in response to the visual attention (114) and understanding of others (115), and who can (at least sometimes) make inferences based on others’ knowledge (67, 68) and expectations (116). However, the code model and the methods commonly used to study animals leave little room for discovering complex or infrequent interactions that involve integrating information across different sources. Recognizing differences in our implicit models of human and animal communication—and the ways in which these models bias our studies—is a necessary step toward credible comparisons between language and animal communication.

### A Path Forward?

It is impossible to eliminate every difference in the way communication is studied in animals and humans, but it is critical to acknowledge and account for theoretical and methodological differences when comparing abilities between species. To draw accurate comparisons between the communication systems and underlying cognitive abilities of humans and animals, animal communication studies must expand beyond searching for code-like signals with predictable behavioral responses.

Scholars studying great ape communication have recently begun to break away from the code model by introducing a range of new methods, mainly adapted from studies of human interaction. Some have introduced analyses of pragmatics, focusing on the ways context can inflect meaning (94, 117); others allow for greater signal ambiguity, so that each signal does not need to have a single typical response to be included in analyses (82). Some have begun to apply analytic frames from the field of conversation analysis to primate gesture, shifting the focus from the signaling individual to the interacting dyad (95–97). Others have argued that ape gesture researchers need to be bolder in their research questions and conclusions, urging scholars to more fully explore the types of communicative inferences apes can make rather than stopping at demonstrations of intentional signaling (118). Still others have begun developing computer-vision and machine-learning methods to circumvent human biases in the categorization of gesture forms or meanings (119). Finally, a few scholars have begun to use analytical frameworks from animal communication to study human interaction [e.g., to study gestures of human infants (120)]. These emerging lines of work have found much greater continuity between animal and human communication than traditional studies. However, most studies of animal communication remain constrained in both imagination and design by the limitations of the code model.

Current work on ape gesture provides some examples for how animal communication research might expand beyond the code model. This echoes an earlier shift in the field—also led by work on ape gesture—toward considering the intentionality of primate signals (e.g., refs. 121, 122). However, many of the more flexible approaches to ape gesture draw directly on methods from studies of human interaction (e.g., conversation analysis and pragmatics). Some of these methods would take significant work to apply to species more distantly related to humans; modifying them to fit different social structures, body plans, perceptual systems, and environments demands added care to avoid stretching definitions and categories past the point of useful comparison. Methods adapted from conversation analysis seem to be among the most directly applicable across a wide range of species (96, 123–125). This is probably because conversation analysis began as a framework to study the underlying rules of human interaction from patterns of external behavior, much as is done with animals (126). Analyses of several aspects of interaction, including turn-taking and communicative repair, have been usefully applied to both animal and human data with only minor modifications (96, 127). The focus of conversation analysis on externally observable behavior helps it transfer between species, but its backgrounding of cognitive processes limits its utility in demonstrating abilities like ostension and inference (128). A wider sea change is needed in the ways researchers approach the communication and minds of animals. Here are two possibilities:

1. Inclusion-by-association: One small but potentially impactful change would be for scholars to grant animals the same generous assumption that they do preverbal human children: that ambiguous behaviors directed toward others in the context of known communicative acts are probably also communicative. This assumption would greatly expand the types of exchanges that are included in studies of animal communication. Inclusion-by-association is common practice in interpretation of preverbal infant behavior, but it is not applied to animals. Instead, animal research typically engages in exclusion-by-association. For example, if an animal behavior occurs alongside signals or behaviors typically found in the context of play, it will often be glossed as play and excluded from further analyses of signal meaning (81, 129). Taking a page from studies of human language development, I propose inclusion-by-association rather than exclusion-by-association.
2. Communicative play: Researchers could also ask whether animals engage in communicative play. Animal play can be viewed as an opportunity for rich inference and potentially for pretense, as individuals signal to others that their actions should not be taken literally (130–132). Some types of play even display game-like qualities involving role reversal and hierarchical rules (116, 133). Despite considerable interest in animal play, communicative play is not often studied. Researchers could start with known signals and ask how animals use those signals in nonstandard ways. They could also conduct qualitative analyses of rare interactions. For example, a researcher might observe animals sometimes producing signals in nonsocial contexts; pairing signals with actions that are not obviously communicative; or using signals in nontypical ways during social play. These nonstandard uses would likely be missed or excluded by current protocols for studying animal communication, but they are similar to behaviors in human children that are known to be important in communicative development. One type of communicative play—babbling—has been documented in animal species that learn to produce their vocalizations. Many species of birds, some species of bats and marine mammals, and at least one species of marmosets go through periods of vocal rehearsal early in development characterized by playful exploration of the acoustic space (134–139). This suggests that there may be other types of communicative play to be found outside of typical signaler-receiver information exchanges.

Opening up studies of animal communication to “messy” signaling would not require that researchers abandon the strict definitions and protocols that ensure interobserver reliability and replicability. Nor would it involve an “anything goes” approach to building a dataset. However, by systematically exploring instances where signals are used in ways that do not fit the expectations and assumptions of the code model, researchers may discover that animals can use a range of semiotic resources in indirect or novel ways.

Studies showing code-like contingencies between signals and responses have been crucial for demonstrating that animal signals have meanings and are not purely reflexes. The proposed methodological shifts toward considering animal communication outside of simple signal-response pairs would not invalidate or discount the contributions of studies following the code model. New, more flexible methods build on findings from code-model analyses, using known codes to anchor or contextualize ambiguous or rare behaviors. By acknowledging historic differences in the ways scholars study animals and humans—and incorporating new approaches that break away from primarily searching for codes—we may find that animal communication and human language are largely characterized by differences of degree rather than kind.

## Data Availability

There are no data underlying this work.

## Footnotes

^*^Cultural transmission does not play a substantial role in the acquisition of either vocal or gestural communication in non-human primates (140–145). Primates, like many other species, do develop cultural traditions in other domains (146).

## References

[1] D. Sperber, D. Wilson, Relevance: Communication and Cognition (Blackwell, 1995).

[2] G. Origgi, D. Sperber, “Evolution, communication and the proper function of language” in Evolution and the Human Mind: Language, Modularity and Social Cognition, P. Carruthers, A. Chamberlain, Eds. (Cambridge University Press, 2000), pp. 140–169.

[3] M. Tomasello, Origins of human Communication (MIT press, 2010).

[4] C. Darwin, The Expression of the Emotions in Man and Animals (Cambridge University Press, 1872), pp. 364–393. The portable Darwin (1993).

[5] C. F. Hockett, The origin of speech. Sci. Am. **203**, 89–96 (1960). 14402211

[6] C. K. Catchpole, P. J. B. Slater, Bird Song: Biological Themes and Variations (Cambridge University Press, 2003).

[7] E. C. Garland, P. K. McGregor, Cultural transmission, evolution, and revolution in vocal displays: Insights from bird and whale song. Front. Psychol. **11**, 544929 (2020). 33132953 10.3389/fpsyg.2020.544929 PMC7550662

[8] J. Hyland Bruno, E. D. Jarvis, M. Liberman, O. Tchernichovski, Birdsong learning and culture: Analogies with human spoken language. Annu. Rev. Linguist. **7**, 449–472 (2021).

[9] P. J. Slater, The cultural transmission of bird song. Trends Ecol. Evol. **1**, 94–97 (1986). 21227788 10.1016/0169-5347(86)90032-7

[10] V. B. Deecke, J. K. Ford, P. Spong, Dialect change in resident killer whales: Implications for vocal learning and cultural transmission. Anim. Behav. **60**, 629–638 (2000). 11082233 10.1006/anbe.2000.1454

[11] V. M. Janik, M. Knörnschild, Vocal production learning in mammals revisited. Philos. Trans. R. Soc. Lond. B Biol. Sci. **376**, 20200244 (2021). 34482736 10.1098/rstb.2020.0244 PMC8419569

[12] J. H. Poole, P. L. Tyack, A. S. Stoeger-Horwath, S. Watwood, Elephants are capable of vocal learning. Nature **434**, 455–456 (2005). 15791244 10.1038/434455a

[13] D. T. Blumstein, The evolution of functionally referential alarm communication: Multiple adaptations; multiple constraints. Evol. Commun. **3**, 135–147 (1999).

[14] S. A. Gill, A.M.-K. Bierema, On the meaning of alarm calls: A review of functional reference in avian alarm calling. Ethology **119**, 449–461 (2013).

[15] J. P. Morford, S. Goldin-Meadow, From here and now to there and then: The development of displaced reference in homesign and English. Child Dev. **68**, 420–435 (1997). 9249958

[16] T. Suddendorf, M. C. Corballis, The evolution of foresight: What is mental time travel, and is it unique to humans? Behav. Brain Sci. **30**, 299–313; discussion 313–351 (2007). 17963565 10.1017/S0140525X07001975

[17] H. Lyn , Apes communicate about absent and displaced objects: Methodology matters. Anim. Cogn. **17**, 85–94 (2014). 23681052 10.1007/s10071-013-0640-0 PMC3818454

[18] N. J. Mulcahy, J. Call, Apes save tools for future use. Science **312**, 1038–1040 (2006). 16709782 10.1126/science.1125456

[19] C. R. Raby, D. M. Alexis, A. Dickinson, N. S. Clayton, Planning for the future by western scrub-jays. Nature **445**, 919–921 (2007). 17314979 10.1038/nature05575

[20] M. Osvath, H. Osvath, Chimpanzee (*Pan troglodytes*) and orangutan (*Pongo abelii*) forethought: Self-control and pre-experience in the face of future tool use. Ani. Cogn. **11**, 661–674 (2008). 10.1007/s10071-008-0157-0 18553113

[21] R. Brown, U. Bellugi, Three processes in the child’s acquisition of syntax. Harv. Educ. Rev. **34**, 133–151 (1964).

[22] N. Chomsky, “Linguistic contributions to the study of mind: Future” in Language and Mind (Cambridge, 1968), pp. 323–364.

[23] D. Bickerton, The supremacy of syntax. Behav. Brain Sci. **10**, 658–659 (1987).

[24] P. Lieberman, Human language and human uniqueness. Lang. Commun. **14**, 87–95 (1994).

[25] R. C. Berwick, K. Okanoya, G. J. L. Beckers, J. J. Bolhuis, Songs to syntax: The linguistics of birdsong. Trends Cogn. Sci. **15**, 113–121 (2011). 21296608 10.1016/j.tics.2011.01.002

[26] W. A. Searcy, J. Soha, S. Peters, S. Nowicki, Long-distance dependencies in birdsong syntax. Proc. Biol. Sci. **289**, 20212473 (2022). 35078357 10.1098/rspb.2021.2473 PMC8790354

[27] K. Payne, R. Payne, Large scale changes over 19 years in songs of humpback whales in Bermuda. Z. Tierpsychol. **68**, 89–114 (2010).

[28] J. C. Mitani, P. Marler, A phonological analysis of male gibbon singing behavior. Behaviour **109**, 20–45 (1989).

[29] E. Balaban, Bird song syntax: Learned intraspecific variation is meaningful. Proc. Natl. Acad. Sci. U.S.A. **85**, 3657–3660 (1988). 3368470 10.1073/pnas.85.10.3657 PMC280273

[30] S. T. Emlen, An experimental analysis of the parameters of bird song eliciting species recognition. Behaviour **41**, 130–171 (1972).

[31] C. E. Taylor, J. T. Brumley, R. W. Hedley, M. L. Cody, Sensitivity of California Thrashers (*Toxostoma redivivum*) to song syntax. Bioacoustics **26**, 259–270 (2017).

[32] C. Crockford, C. Boesch, Call combinations in wild chimpanzees. Behaviour **142**, 397–421 (2005).

[33] K. Zuberbühler, A syntactic rule in forest monkey communication. Anim. Behav. **63**, 293–299 (2002).

[34] R. M. Schassburger, Vocal Communication in the Timber Wolf, Canis lupus: Structure, Motivation, and Ontogeny (Parey Scientific Publication, 1993).

[35] J. G. Robinson, Syntactic structures in the vocalizations of wedge-capped capuchin monkeys, *Cebus olivaceus*. Behaviour **90**, 46–78 (1984).

[36] K. Martin, A. G. Horn, S. J. Hannon, The calls and associated behavior of breeding willow ptarmigan in Canada. Wilson Bull. **107**, 496–509 (1995).

[37] J. Cleveland, C. T. Snowdon, The complex vocal repertoire of the adult cotton-top Tamarin (*Saguinus oedipus oedipus*)1). Z. Tierpsychol. **58**, 231–270 (2010).

[38] K. Arnold, K. Zuberbühler, Semantic combinations in primate calls. Nature **441**, 303–303 (2006). 16710411 10.1038/441303a

[39] K. Arnold, K. Zuberbühler, Meaningful call combinations in a non-human primate. Curr. Biol. **18**, R202–R203 (2008). 18334192 10.1016/j.cub.2008.01.040

[40] P. Schlenker, E. Chemla, K. Arnold, K. Zuberbühler, Pyow-hack revisited: Two analyses of Putty-nosed monkey alarm calls. Lingua **171**, 1–23 (2016).

[41] T. N. Suzuki, D. Wheatcroft, M. Griesser, The syntax–semantics interface in animal vocal communication. Philos. Trans. R. Soc. Lond. B Biol. Sci. **375**, 20180405 (2019). 31735156 10.1098/rstb.2018.0405 PMC6895555

[42] T. N. Suzuki, Semantic communication in birds: Evidence from field research over the past two decades. Ecol. Res. **31**, 307–319 (2016).

[43] P. Schlenker, C. Coye, M. Leroux, E. Chemla, The ABC-D of animal linguistics: Are syntax and compositionality for real? Biol. Rev. Camb. Philos. Soc. **98**, 1142–1159 (2023). 36960599 10.1111/brv.12944

[44] N. M. Kime, A. S. Rand, M. Kapfer, M. J. Ryan, Consistency of female choice in the túngara frog: A permissive preference for complex characters. Anim. Behav. **55**, 641–649 (1998). 9514683 10.1006/anbe.1997.0752

[45] S. Zsebők , Sequential organization of birdsong: Relationships with individual quality and fitness. Behav. Ecol. **32**, 82–93 (2021). 33708006 10.1093/beheco/araa104 PMC7937035

[46] M. D. Hauser, N. Chomsky, W. T. Fitch, The faculty of language: What is it, who has it, and how did it evolve? Science **298**, 1569–1579 (2002). 12446899 10.1126/science.298.5598.1569

[47] T. Q. Gentner, K. M. Fenn, D. Margoliash, H. C. Nusbaum, Recursive syntactic pattern learning by songbirds. Nature **440**, 1204–1207 (2006). 16641998 10.1038/nature04675 PMC2653278

[48] C. A. A. van Heijningen, J. de Visser, W. Zuidema, C. ten Cate, Simple rules can explain discrimination of putative recursive syntactic structures by a songbird species. Proc. Natl. Acad. Sci. U.S.A. **106**, 20538–20543 (2009). 19918074 10.1073/pnas.0908113106 PMC2787117

[49] T. Scott-Phillips, Speaking Our Minds: Why Human Communication is Different, and How Language Evolved to Make It Special (Bloomsbury Publishing, 2014).

[50] T. C. Scott-Phillips, Nonhuman primate communication, pragmatics, and the origins of language. Curr. Anthropol. **56**, 56–80 (2015).

[51] K. Andrews, The Animal Mind: An Introduction to the Philosophy of Animal Cognition (Routledge, 2020).

[52] H. P. Grice, “Logic and conversation” in Syntax and Semantics, Vol. 3, Speech Acts, P. Cole, J. L. Morgan, Eds. (Academic Press, 1975), pp. 41–58.

[53] D. Bar-On, Origins of meaning: Must we “go Gricean”? Mind Lang. **28**, 342–375 (2013).

[54] T. C. Scott-Phillips, Meaning in great ape communication: Summarising the debate. Anim. Cogn. **19**, 233–238 (2016). 26573579 10.1007/s10071-015-0936-3 PMC4701766

[55] R. Moore, Meaning and ostension in great ape gestural communication. Anim. Cogn. **19**, 223–231 (2016). 26223212 10.1007/s10071-015-0905-x

[56] K. E. Graham, C. Wilke, N. J. Lahiff, K. E. Slocombe, Scratching beneath the surface: Intentionality in great ape signal production. Philos. Trans. R. Soc. Lond. B Biol. Sci. **375**, 20180403 (2020). 31735155 10.1098/rstb.2018.0403 PMC6895546

[57] R. J. Planer, Going Dennettian about Gricean communication. Philos. Psychol. 1–24 (2023).

[58] M. Tomasello, A Natural History of Human Thinking (Harvard University Press, 2018).

[59] D. Sperber, D. Wilson, Pragmatics, modularity and mind‐reading. Mind & Language **17**, 3–23 (2002).

[60] B. Hare, J. Call, B. Agnetta, M. Tomasello, Chimpanzees know what conspecifics do and do not see. Anim. Behav. **59**, 771–785 (2000). 10792932 10.1006/anbe.1999.1377

[61] D. L. Cheney, Extent and limits of cooperation in animals. Proc. Natl. Acad. Sci. U.S.A. **108** (Suppl. 2), 10902–10909 (2011). 21690405 10.1073/pnas.1100291108 PMC3131815

[62] G. L. Davidson, N. S. Clayton, New perspectives in gaze sensitivity research. Learn. Behav. **44**, 9–17 (2016). 26582567 10.3758/s13420-015-0204-z

[63] D. Cheney, R. Seyfarth, B. Smuts, Social relationships and social cognition in nonhuman primates. Science **234**, 1361–1366 (1986). 3538419 10.1126/science.3538419

[64] R. W. Byrne, L. A. Bates, Primate social cognition: Uniquely primate, uniquely social, or just unique? Neuron **65**, 815–830 (2010). 20346757 10.1016/j.neuron.2010.03.010

[65] A. G. Rosati, L. R. Santos, B. Hare, “Primate social cognition: Thirty years after Premack and Woodruff” in Primate Neuroethology, M. L. Platt, A. A. Ghazanfar, Eds. (Oxford University Press, 2010), pp. 117–143.

[66] D. L. Cheney, R. M. Seyfarth, How Monkeys See the World: Inside the Mind of Another Species (University of Chicago Press, 2018).

[67] C. Krupenye, F. Kano, S. Hirata, J. Call, M. Tomasello, Great apes anticipate that other individuals will act according to false beliefs. Science **354**, 110–114 (2016). 27846501 10.1126/science.aaf8110

[68] F. Kano, C. Krupenye, S. Hirata, M. Tomonaga, J. Call, Great apes use self-experience to anticipate an agent’s action in a false-belief test. Proc. Natl. Acad. Sci. U.S.A. **116**, 20904–20909 (2019). 31570582 10.1073/pnas.1910095116 PMC6800361

[69] H. M. Wellman, Theory of mind: The state of the art. Eur. J. Dev. Psychol. **15**, 728–755 (2018).

[70] V. Slaughter, Theory of mind in infants and young children: A review. Aust. Psychol. **50**, 169–172 (2015).

[71] D. Premack, G. Woodruff, Does the chimpanzee have a theory of mind? Behav. Brain Sci. **1**, 515–526 (1978).

[72] R. M. Scott, Z. He, R. Baillargeon, D. Cummins, False-belief understanding in 2.5-year-olds: Evidence from two novel verbal spontaneous-response tasks. Dev. Sci. **15**, 181–193 (2012). 22356174 10.1111/j.1467-7687.2011.01103.x PMC3292198

[73] J. de Villiers, The interface of language and theory of mind. Lingua **117**, 1858–1878 (2007). 17973025 10.1016/j.lingua.2006.11.006 PMC2000852

[74] C. E. Shannon, A mathematical theory of communication. Bell Syst. Techn. J. **27**, 379–423 (1948).

[75] M. J. Reddy, “The conduit metaphor” in Metaphor and Thought, A. Ortony Ed. (Cambridge, 1979), pp. 284–310.

[76] S. W. Townsend , Exorcising Grice’s ghost: An empirical approach to studying intentional communication in animals. Biol. Rev. Camb. Philos. Soc. **92**, 1427–1433 (2017). 27480784 10.1111/brv.12289

[77] C. S. Evans, L. Evans, P. Marler, On the meaning of alarm calls: Functional reference in an avian vocal system. Anim. Behav. **46**, 23–38 (1993).

[78] S. A. Gelman, E. M. Markman, Young children’s inductions from natural kinds: The role of categories and appearances. Child Dev. **58**, 1532–1541 (1987). 3691200

[79] P. C. Quinn, Category representation in young infants. Curr. Dir. Psychol. Sci. **11**, 66–70 (2002).

[80] E. A. Cartmill, R. W. Byrne, Semantics of primate gestures: Intentional meanings of orangutan gestures. Anim. Cogn. **13**, 793–804 (2010). 20563619 10.1007/s10071-010-0328-7

[81] C. Hobaiter, R. W. Byrne, The meanings of chimpanzee gestures. Curr. Biol. **24**, 1596–1600 (2014). 24998524 10.1016/j.cub.2014.05.066

[82] C. Hobaiter, K. E. Graham, R. W. Byrne, Are ape gestures like words? Outstanding issues in detecting similarities and differences between human language and ape gesture. Philos. Trans. R. Soc. Lond. B Biol. Sci. **377**, 20210301 (2022). 35934962 10.1098/rstb.2021.0301 PMC9358316

[83] R. M. Seyfarth, D. L. Cheney, Signalers and receivers in animal communication. Annu. Rev. Psychol. **54**, 145–173 (2003). 12359915 10.1146/annurev.psych.54.101601.145121

[84] M. Tomasello, J. Call, K. Nagell, R. Olguin, M. Carpenter, The learning and use of gestural signals by young chimpanzees: A trans-generational study. Primates **35**, 137–154 (1994).

[85] S. Pika, K. Liebal, M. Tomasello, Gestural communication in young gorillas (*Gorilla gorilla*): Gestural repertoire, learning, and use. Am. J. Primatol. **60**, 95–111 (2003). 12874841 10.1002/ajp.10097

[86] K. Liebal, S. Pika, M. Tomasello, Gestural communication of orangutans (*Pongo pygmaeus*). Gesture (Amst.) **6**, 1–38 (2006).

[87] F. X. Plooij, “The behavioral development of free-living chimpanzee babies and infants” in Monographs on Infancy (Bloomsbury Academic Press, 1984), vol. 4, p. 207.

[88] F. X. Plooij, “Some basic traits of language in wild chimpanzees?” In Action, Gesture and Symbol, A. Lock, Ed. (1978).

[89] J. Van Lawick-Goodall, The behaviour of free-living chimpanzees in the Gombe stream reserve. Anim. Behav. Monogr. **1**, 161–311, IN1–IN12 (1968).

[90] A. S. Pollick, F. B. M. de Waal, Ape gestures and language evolution. Proc. Natl. Acad. Sci. U. S. A. **104**, 8184–8189 (2007). 17470779 10.1073/pnas.0702624104 PMC1876592

[91] M. A. Arbib, K. Liebal, S. Pika, Primate vocalization, gesture, and the evolution of human language. Curr. Anthropol. **49**, 1053–1076 (2008). 19391445 10.1086/593015

[92] R. W. Byrne , Great ape gestures: Intentional communication with a rich set of innate signals. Anim. Cogn. **20**, 755–769 (2017). 28502063 10.1007/s10071-017-1096-4 PMC5486474

[93] E. Genty, T. Breuer, C. Hobaiter, R. W. Byrne, Gestural communication of the gorilla (*Gorilla* *gorilla*): Repertoire, intentionality and possible origins. Anim. Cogn. **12**, 527–546 (2009). 19184669 10.1007/s10071-009-0213-4 PMC2757608

[94] M. Bohn, K. Liebal, L. Oña, M. H. Tessler, Great ape communication as contextual social inference: A computational modelling perspective. Philos. Trans. R. Soc. Lond. B Biol. Sci. **377**, 20210096 (2022). 35876204 10.1098/rstb.2021.0096 PMC9310183

[95] E. Genty , How apes get into and out of joint actions: Shared intentionality as an interactional achievement. Interact. Stud. **21**, 353–386 (2020).

[96] R. Heesen, M. Fröhlich, C. Sievers, M. Woensdregt, M. Dingemanse, Coordinating social action: A primer for the cross-species investigation of communicative repair. Philos. Trans. R. Soc. Lond. B Biol. Sci. **377**, 20210110 (2022). 35876201 10.1098/rstb.2021.0110 PMC9310172

[97] L. Mondada, A. Meguerditchian, Sequence organization and embodied mutual orientations: Openings of social interactions between baboons. Philos. Trans. R. Soc. Lond. B Biol. Sci. **377**, 20210101 (2022). 35876203 10.1098/rstb.2021.0101 PMC9310171

[98] K. E. Graham, T. Furuichi, R. W. Byrne, Context, not sequence order, affects the meaning of bonobo (*Pan paniscus*) gestures. Gesture **19**, 335–364 (2020).

[99] K. E. Graham, G. Badihi, A. Safryghin, C. Grund, C. Hobaiter, A socio-ecological perspective on the gestural communication of great ape species, individuals, and social units. Ethol. Ecol. Evol. **34**, 235–259 (2022). 35529671 10.1080/03949370.2021.1988722 PMC9067943

[100] E. A. Schegloff, G. Jefferson, H. Sacks, The preference for self-correction in the organization of repair in conversation. Language **53**, 361–382 (1977).

[101] J. Heritage, Garfinkel and Ethnomethodology (John Wiley & Sons, 2013).

[102] R. B. Church, S. Kelly, D. Holcombe, Temporal synchrony between speech, action and gesture during language production. Lang. Cogn. Neurosci. **29**, 345–354 (2014).

[103] N. Esteve-Gibert, P. Prieto, F. Pons, Nine-month-old infants are sensitive to the temporal alignment of prosodic and gesture prominences. Infant Behav. Dev. **38**, 126–129 (2015). 25656953 10.1016/j.infbeh.2014.12.016

[104] H. R. Bosker, D. Peeters, Beat gestures influence which speech sounds you hear. Proc. Biol. Sci. **288**, 20202419 (2021). 33499783 10.1098/rspb.2020.2419 PMC7893284

[105] R. I. Mayberry, J. Jaques, “Gesture production during stuttered speech: Insights into the nature of gesture-speech integration” in Language and Gesture, D. McNeill, Ed. (Cambridge University Press, Cambridge, 2000), pp. 199–214.

[106] M. Graziano, M. Gullberg, When speech stops, gesture stops: Evidence from developmental and crosslinguistic comparisons. Front. Psychol. **9**, 879 (2018). 29910761 10.3389/fpsyg.2018.00879 PMC5992892

[107] J. Cassell, D. McNeill, K.-E. McCullough, Speech-gesture mismatches: Evidence for one underlying representation of linguistic and nonlinguistic information. Pragmat. Cogn. **7**, 1–34 (1999).

[108] S. Goldin-Meadow, M. A. Singer, From children’s hands to adults’ ears: Gesture’s role in the learning process. Dev. Psychol. **39**, 509–520 (2003). 12760519 10.1037/0012-1649.39.3.509

[109] A. Kendon, “Gesticulation and speech: Two aspects of the process of utterance” in The Relationship of Verbal and Nonverbal Communication, M. Key, Ed. (Mouton, 1980), pp. 207–227.

[110] D. McNeill, Hand and Mind. What Gestures Reveal about Thought (University of Chicago Press, Chicago, 1992).

[111] D. McNeill, S. D. Duncan, "Growth points in thinking-for-speaking" in Language and Gesture (Cambridge University Press, Cambridge, 2000), **Vol. 1987**, pp. 141–161.

[112] E. A. Cartmill, Gesture. Ann. Rev. Anthropol. **51**, 455–73 (2022).

[113] C. Goodwin, Co-Operative Action (Cambridge University Press, 2018).

[114] K. Liebal, J. Call, M. Tomasello, S. Pika, To move or not to move: How apes adjust to the attentional state of others. Interact. Stud. **5**, 199–219 (2004).

[115] E. A. Cartmill, R. W. Byrne, Orangutans modify their gestural signaling according to their audience’s comprehension. Curr. Biol. **17**, 1345–1348 (2007). 17683939 10.1016/j.cub.2007.06.069

[116] J. Eckert, S. L. Winkler, E. A. Cartmill, Just kidding: The evolutionary roots of playful teasing. Biol. Lett. **16**, 20200370 (2020). 32961087 10.1098/rsbl.2020.0370 PMC7532725

[117] C. Sievers, T. Gruber, Reference in human and non-human primate communication: What does it take to refer? Anim. Cogn. **19**, 759–768 (2016). 26971953 10.1007/s10071-016-0974-5

[118] E. Warren, J. Call, Inferential communication: Bridging the gap between intentional and ostensive communication in non-human primates. Front. Psychol. **12**, 718251 (2021). 35095633 10.3389/fpsyg.2021.718251 PMC8795877

[119] C. Wiltshire , DeepWild: Application of the pose estimation tool DeepLabCut for behaviour tracking in wild chimpanzees and bonobos. J. Anim. Ecol. **92**, 1560–1574 (2023), 10.1111/1365-2656.13932. 37165474

[120] V. Kersken, J.-C. Gómez, U. Liszkowski, A. Soldati, C. Hobaiter, A gestural repertoire of 1- to 2-year-old human children: In search of the ape gestures. Anim. Cogn. **22**, 577–595 (2019). 30196330 10.1007/s10071-018-1213-z PMC6647402

[121] C. Crockford, R. M. Wittig, R. Mundry, K. Zuberbühler, Wild chimpanzees inform ignorant group members of danger. Curr. Biol. **22**, 142–146 (2012). 22209531 10.1016/j.cub.2011.11.053

[122] A. M. Schel, S. W. Townsend, Z. Machanda, K. Zuberbühler, K. E. Slocombe, Chimpanzee alarm call production meets key criteria for intentionality. PLoS One **8**, e76674 (2013). 24146908 10.1371/journal.pone.0076674 PMC3797826

[123] D. M. Logue, T. Stivers, Squawk in interaction: A primer of conversation analysis for students of animal communication. Behaviour **149**, 1283–1298 (2012).

[124] M. Fröhlich, Taking turns across channels: Conversation-analytic tools in animal communication. Neurosci. Biobehav. Rev. **80**, 201–209 (2017). 28501552 10.1016/j.neubiorev.2017.05.005

[125] F. Abreu, S. Pika, Turn-taking skills in mammals: A systematic review into development and acquisition. Front. Ecol. Evol. **10**, 987253 (2022).

[126] C. Goodwin, J. Heritage, Conversation analysis. Annu. Rev. Anthropol. **19**, 283–307 (1990).

[127] K. D. Rivera-Cáceres, E. Quirós-Guerrero, M. Araya-Salas, C. N. Templeton, W. A. Searcy, Early development of vocal interaction rules in a duetting songbird. R. Soc. Open Sci. **5**, 171791 (2018). 29515888 10.1098/rsos.171791 PMC5830777

[128] J. Potter, D. Edwards, “Conversation analysis and psychology” in The Handbook of Conversation Analysis, J. Sidnell, T. Stivers, Eds. (John Wiley & Sons, 2012), pp. 701–725.

[129] O. M. J. Adang, Teasing in young chimpanzees. Behaviour **88**, 98–121 (1984).

[130] M. Bekoff, J. A. Byers, Animal Play: Evolutionary, Comparative and Ecological Perspectives (Cambridge University Press, 1998).

[131] M. Bekoff, C. Allen, “Intentional communication and social play: How and why animals negotiate and agree to play” in Animal Play: Evolutionary, Comparative and Ecological Perspectives, M. Bekoff, J. A. Byers, Eds. (Cambridge University Press, 1998), pp. 97–114.

[132] E. Palagi , Rough-and-tumble play as a window on animal communication. Biol. Rev. Camb. Philos. Soc. **91**, 311–327 (2016). 25619897 10.1111/brv.12172

[133] A. Mielke, S. Carvalho, Chimpanzee play sequences are structured hierarchically as games. PeerJ **10**, e14294 (2022). 36411837 10.7717/peerj.14294 PMC9675342

[134] A. A. Fernandez, L. S. Burchardt, M. Nagy, M. Knörnschild, Babbling in a vocal learning bat resembles human infant babbling. Science **373**, 923–926 (2021). 34413237 10.1126/science.abf9279

[135] D. Cazau, O. Adam, J. T. Laitman, J. S. Reidenberg, Understanding the intentional acoustic behavior of humpback whales: A production-based approach. J. Acoust. Soc. Am. **134**, 2268–2273 (2013). 23967956 10.1121/1.4816403

[136] S. M. Ter Haar , Cross-species parallels in babbling: Animals and algorithms. Philos. Trans. R. Soc. Lond. B Biol. Sci. **376**, 20200239 (2021). 34482727 10.1098/rstb.2020.0239 PMC8419573

[137] A. Margaret Elowson, C. T. Snowdon, C. Lazaro-Perea, ‘Babbling’ and social context in infant monkeys: Parallels to human infants. Trends Cogn. Sci. **2**, 31–37 (1998). 21244960 10.1016/s1364-6613(97)01115-7

[138] A. L. Pistorio, B. Vintch, X. Wang, Acoustic analysis of vocal development in a New World primate, the common marmoset (*Callithrix jacchus*). J. Acoust. Soc. Am. **120**, 1655–1670 (2006). 17004487 10.1121/1.2225899

[139] B. McCowan, D. Reiss, Whistle contour development in captive-born infant bottlenose dolphins (*Tursiops truncatus*): Role of learning. J. Comp. Psychol. **109**, 242–260 (1995).

[140] K. J. Hayes, C. Hayes, The intellectual development of a home-raised chimpanzee. Proc. Am. Philos. Soc. **95**, 105–109 (1951).

[141] W. N. Kellogg, Communication and Language in the Home-Raised Chimpanzee. Science **162**, 423–427 (1968). 5683047 10.1126/science.162.3852.423

[142] N. P. Desai, P. Fedurek, K. E. Slocombe, M. L. Wilson, Chimpanzee pant-hoots encode individual information more reliably than group differences. Am. J. Primatol. **84**, e23430 (2022). 36093564 10.1002/ajp.23430 PMC9786991

[143] M. J. Owren, J. A. Dieter, R. M. Seyfarth, D. L. Cheney, “Food” calls produced by adult female rhesus (*Macaca mulatta*) and Japanese (*M. fuscata*) macaques, their normally-raised offspring, and offspring cross-fostered between species. Behaviour **120**, 218–231 (1992).

[144] G. Badihi , Dialects in leaf-clipping and other leaf-modifying gestures between neighbouring communities of East African chimpanzees. Sci. Rep. **13**, 147 (2023). 36604445 10.1038/s41598-022-25814-x PMC9814361

[145] E. A. Cartmill, C. Hobaiter, Developmental perspectives on primate gesture: 100 years in the making. Anim. Cogn. **22**, 453–459 (2019). 31278622 10.1007/s10071-019-01279-w

[146] A. Whiten, The burgeoning reach of animal culture. Science **372**, eabe6514 (2021). 33795431 10.1126/science.abe6514

